# Grade follicles transcriptional profiling analysis in different laying stages in chicken

**DOI:** 10.1186/s12864-022-08728-w

**Published:** 2022-07-07

**Authors:** Tiantian Sun, Cong Xiao, Zhuliang Yang, Jixian Deng, Xiurong Yang

**Affiliations:** College of Animal Science and Technology, Guangxi University, Nanning, 530004 China

**Keywords:** Nandan-Yao Domestic chicken, Follicular development, RNA-Seq, Transcriptome

## Abstract

During follicular development, a series of key events such as follicular recruitment and selection are crucially governed by strict complex regulation. However, its molecular mechanisms remain obscure. To identify the dominant genes controlling chicken follicular development, the small white follicle (SWF), the small yellow follicle (SYF), and the large yellow follicle (LYF) in different laying stages (W22, W31, W51) were collected for RNA sequencing and bioinformatics analysis. There were 1866, 1211, and 1515 differentially expressed genes (DEGs) between SWF and SYF in W22, W31, and W51, respectively. 4021, 2295, and 2902 DEGs were respectively identified between SYF and LYF in W22, W31, and W51. 5618, 4016, and 4809 DEGs were respectively identified between SWF and LYF in W22, W31, and W51. Gene Ontology (GO) and Kyoto Encyclopedia of Genes and Genomes (KEGG) analysis indicated that extracellular matrix, extracellular region, extracellular region part, ECM-receptor interaction, collagen extracellular matrix, and collagen trimer were significantly enriched (*P* < 0.05). Protein–protein interaction analysis revealed that *COL4A2*, *COL1A2*, *COL4A1*, *COL5A2*, *COL12A1*, *ELN*, *ALB*, and *MMP10* might be key candidate genes for follicular development in chicken. The current study identified dominant genes and pathways contributing to our understanding of chicken follicular development.

**Supplementary Information:**

The online version contains supplementary material available at 10.1186/s12864-022-08728-w.

## Introduction

In poultry breeds, high-efficiency follicular development means huge economic output for the egg industry. Follicles at different stages exist in the ovary of sexually mature hens, and a hierarchical system is formed in the ovary according to different functions and sizes: Pre-hierarchical follicle and hierarchical follicle (also known as pre-ovulatory follicle) [1]. Once ovulated, a new follicle is selected from the pre-hierarchal cohort to enter the hierarchical stage [2]. The development of follicles is crucially governed by strict intrinsic complex regulation [3]. During follicular development, a series of key events such as gene transcription and protein expression occur in series and are governed by specific gene expression, which is an intrinsic factor regulating follicular recruitment, selection, and apoptosis of follicles [4].

Over the past decade, RNA sequencing (RNA-seq) has become an indispensable tool for transcriptome analysis. RNA-seq is often used for analyzing differential expression genes [5]. RNA-seq has been widely applied to a variety of organisms such as Pigs [6], cattle [7], chickens [8], goats [9], deer [10], and mice [11]. Transcriptome studies have involved various traits such as egg quality [12], meat quality [13], genetic diversity [14], poultry disease screening [15], human aging [16], skeletal muscle development [17], growth [18].

The Nandan-Yao domestic chicken is a native breed in China. It has the characteristics of coarse food resistance, strong foraging ability, delicate and delicious meat, but its performance in egg production is low. In this study, RNA-seq and bioinformatics analysis were performed to identify the differentially expressed genes and pathways between different follicles in different laying stages to reveal the molecular mechanisms of follicular development.

## Materials and methods

### Ethics statement

All experimental and sample collection procedures were approved by the Institutional Animal Care and Use Committee (IACUC) of the College of Animal Science and Technology of Guangxi University (Guangxi, China), with approval number GXU2018-058.

### Separation of follicles

Nandan-Yao domestic hens (*Gallus gallus*) purchased from Guangxi Guigang Gangfeng Agriculture and Husbandry Co. Ltd, laying continuously 3 eggs, were used in this study. Hens in early laying (22 weeks old, with a mean body weight of 1.67 ± 0.02 kg), peak laying (31 weeks old, with a mean body weight of 1.88 ± 0.06 kg), and late laying (51 weeks old, with a mean body weight of 2. 28 ± 0.08 kg) were selected for ovarian follicle sampling (*n* = 4). Follicles within the ovary were classified as small white follicles (SWF, 2–4 mm in diameter), large white follicles (LWF, 4–6 mm in diameter), small yellow follicles (SYF, 6–8 mm in diameter), and large yellow follicles (12–40 mm, named F5, F4, F3, F2, and F1, respectively) [19, 20]. SWFs, SYFs, and LYFs (F1, F2, F3) were collected for RNA extraction. The follicles were washed in PBS to remove the yolk.

### Total RNA extraction

The total RNA was extracted from SWF, SYF, and LYF using TRIzol reagent (Invitrogen Life Technologies, USA) according to the manufacturer's instructions. RNA integrity was monitored on 1% agarose gels. RNA concentration was checked using the UV–Vis Spectrophotometer Q5000 (Quawell, USA).

### RNA sequencing and quality control

The cDNA libraries were constructed and sequenced following the manufacturer’s standard procedures on an Illumina HiSeq 2500 (Illumina, San Diego, CA, USA) in Novogene Bioinformatics Technology Co., Ltd., Beijing, China. Raw reads of FASTQ format were processed with trim galore [21]. To obtain the clean reads, the sequence with low quality including adaptor sequences, quality score < 20, and N base rate of raw reads > 10% were removed. The Q20 scores, GC content, and sequence duplication levels of the clean data were calculated using FastQC [22].

### RNA-Seq analysis

Reference genome and gene model annotation files were downloaded from the genome website (http://ftp.ensembl.org/pub/release-102/gtf/gallus_gallus/, http://ftp.ensembl.org/pub/release-102/fasta/gallus_gallus/dna/). The clean reads were mapped to the chicken reference genome using Hisat2v2.1.0 [23, 24]. The stringtiev2.1.1 was then used to annotate the transcripts [25]. The differential expressed genes between samples were identified using the DESeq2 R package (1.18.0) [26]. The *P*-value < 0.05 and |foldchange|> 2 were used as the criteria of significance. GO term and KEGG pathway analyses of coding genes were performed by the R package clusterProfiler 3.14.3 [27–30]. Both GO terms and KEGG pathways with corrected *P*-adjust < 0.05 were considered to be significantly enriched. The STRING (Franceschini et al., 2013) database was used to explore the interaction between DEGs. A confidence score > 0.9 was defined as valid.

### Validation of RNA-Seq

RNA was reverse transcribed into cDNA using RT Reagent Kit (Takara, Dalian, China). Primer sequences of target and reference genes were shown in Supplemental table 1. QRT-PCR was carried out using SYBR Green Supermix kit (Takara, Dalian, China) in Bio-RAD CFX96 Real Time Detection system. The expression of *β-actin* was used to correct the gene expression data. The 2^−ΔΔCT^ method was used to analyze the QRT-PCR data and calculate relative expression.

## Results

### Transcriptome data

As shown in Supplementary table 2, 18,911,563 to 34,680,085 clean reads per sample were obtained after quality control. The average GC content of all samples was 52.54%. The average mapped rate was 92.38% comparing clean reads with the reference genome. For all samples, at least 96.75% of the reads were equal to or exceeded Q20.

### Analysis of differential expressed genes

**Fig. 1:**
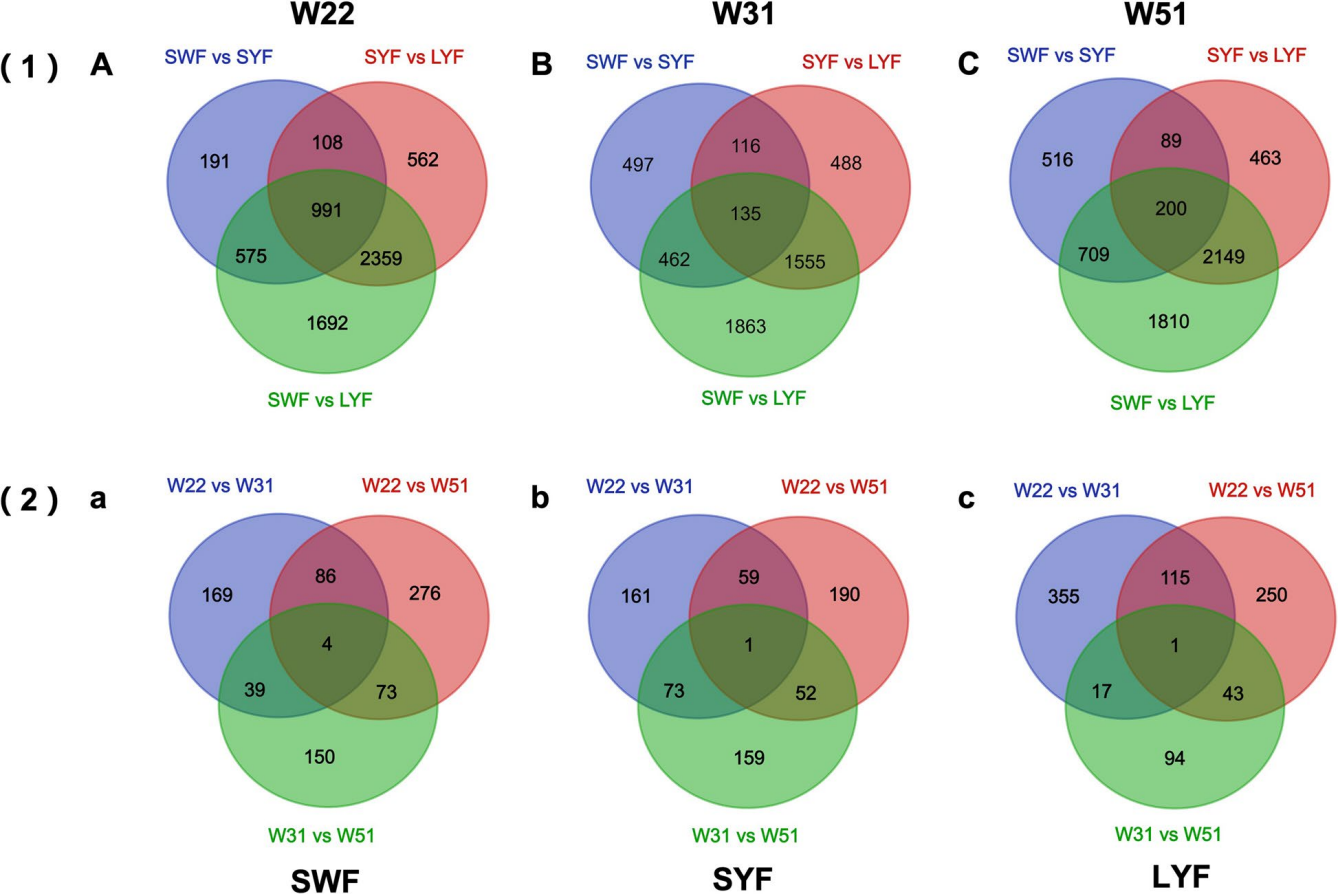
Venn diagram of differentially expressed genes. (1) represents the Venn diagram of differentially expressed genes between different follicles at the same age. A, B, and C represent W21, W31, and W51, respectively. (2) represents the Venn diagram of differentially expressed genes in follicles of the same grade at different ages. a, b, and c represent SWF, SYF, and LYF, respectively. Abbreviations: SWF: small white follicle; SYF: small yellow follicle; LYF: large yellow follicle

At W22, 1866, 4021, and 5618 DEGs were respectively identified between SWF and SYF, SYF and LYF, SWF and LYF (Fig. 1A). At W31,1211, 2295 and 4016 DEGs were respectively identified between SWF and SYF, SYF and LYF, SWF and LYF (Fig. 1B). At W51, the number of DEGs between SWF and SYF, SYF and LYF, SWF and LYF were 1515, 2902, and 4809, respectively (Fig. 1C). As shown in Fig. 1a, 299, 440, and 267 DEGs were respectively obtained between W22 and W31, W22 and W51, W31 and W51 of SWF. In SYF, 295, 303, and 286 DEGs were respectively identified between W22 and W31, W22 and W51, W31 and W51 (Fig. 1b). In LYF, the number of DEGs between W22 and W31, W22 and W51, W31 and W51 were 488, 156, and 410, respectively (Fig. 1c).

### GO and KEGG analysis for DEGs

**Fig. 2:**
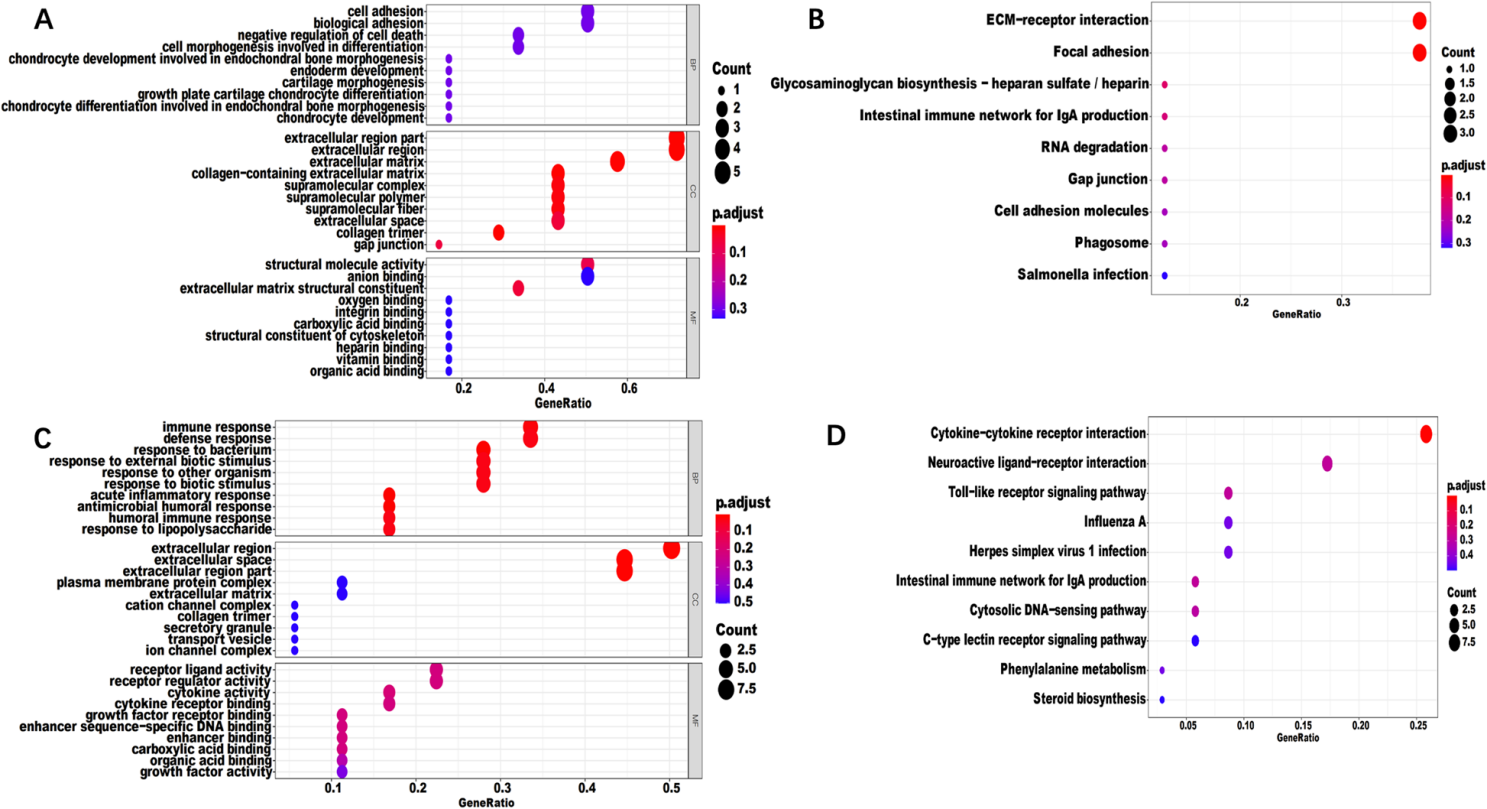
GO and KEGG enrichment analysis of DEGs. **A** and **B** represent GO enrichment and KEGG analysis of DEGs in the W51 intersection, respectively. **C** and **D** represent GO enrichment and KEGG analysis of DEGs in SWF intersection, respectively

Functional enrichment analysis was performed on DEGs of W51 intersection of SWF vs SYF, SYF vs LYF, SWF vs LYF (Fig. 2A, B). GO analysis indicated that differentially expressed genes were enriched in 8 items, including extracellular matrix, collagen-containing extracellular matrix, extracellular region part, extracellular region, collagen trimer, supramolecular complex, supramolecular polymer, supramolecular fiber (Supplementary table 3). KEGG analysis of differentially expressed mRNAs significantly enriched the ECM-receptor interaction and the Focal adhesion pathway (Supplementary table 4).

Functional enrichment analysis was carried out for the intersection of DEGs of W22 vs W31, W22 vs W51, W31 vs W51 in SWF (Fig. 2C, D). The results showed that 17 items were significantly enriched in GO analysis (Supplementary table 5), such as extracellular region, extracellular region part, extracellular space, etc., and cytokine-cytokine receptor interaction was significantly enriched in KEGG analysis (Supplementary table 6).

### Integration of PPI network

**Fig. 3:**
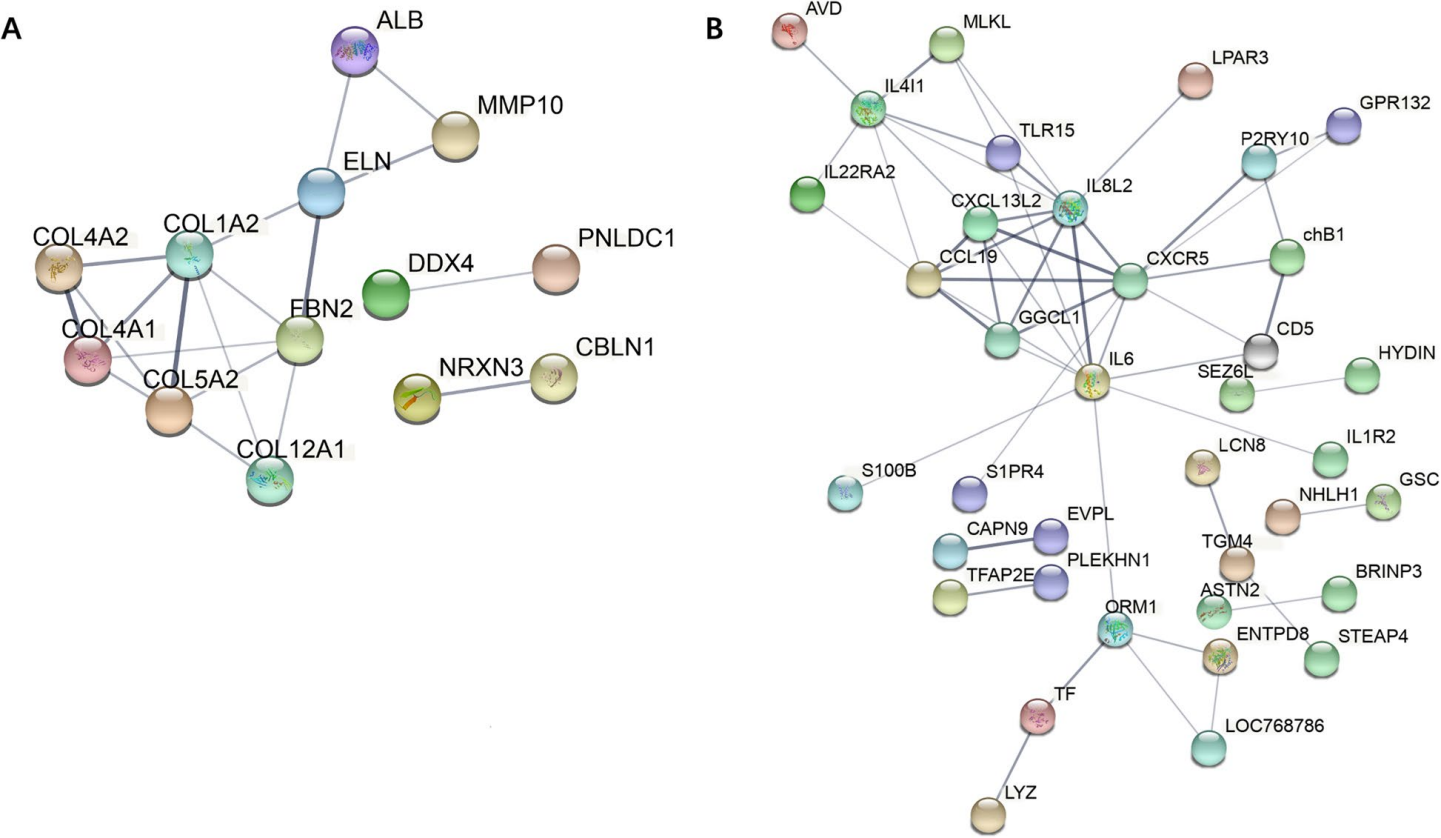
The protein–protein interaction (PPI) network of DEGs. (**A**) PPI analysis of DEGs in the W51 intersection. (**B**) PPI analysis of DEGs in the SWF instersection. Line thickness indicates the strength of data support

To reveal how these DEGs may interact, protein–protein interaction analyses were carried out based on the STRING database. The DEG network interaction analysis of W51 and SWF is shown in Fig. 3. The DEG network of W51 contains 13 genes, while the DEG network of SWF contains 37 genes. These genes may play an important regulatory role in the laying process.

### Validation of RNA-seq

**Fig. 4:**
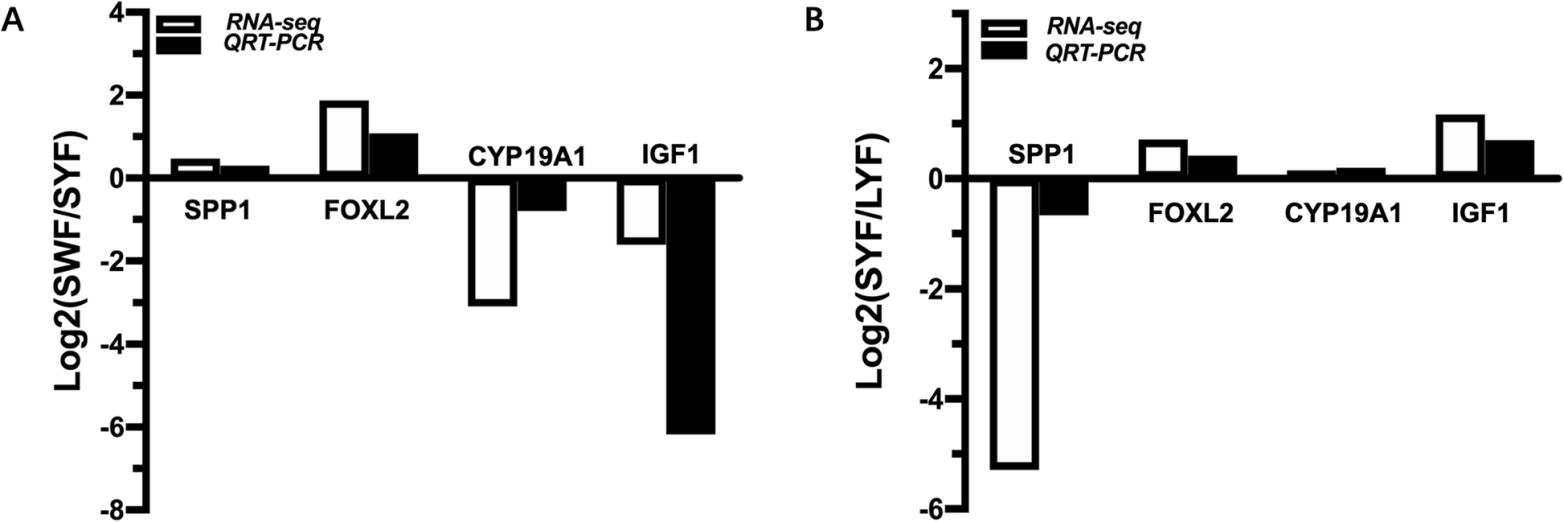
RNA-Seq validation using QRT-PCR. 4 DEGs (*CYP19A1*, *FOXL2*, *IGF1*, *SPP1*) were selected to test the accuracy of RNA sequencing. (**A**) Validation in the SWF and SYF at W22. (**B**) Validation in the SYF and LYF at W22. *N* = 6

To verify our RNA-seq data, we selected 4 genes (*CYP19A1*, *FOXL2*, *IGF1*, *SPP1*) related to follicular development for QRT-PCR analysis (Fig. 4). The results showed that the differentially expressed genes had the same expression trends in QRT-PCR and RNA-seq, which validated their accuracy.

## Discussion

Follicular development is a complex physiological process, regulated by diverse genes and endocrine hormones [3]. Previous studies revealed the effect of *GPR12* [20], *GREM1* [31], *BMP4* [32], *RAC1* [33], *FOXO3* [34], *bFGF* [35, 36], Melatonin [37, 38], *CSAL1* and *CSAL3* [39] on follicular development. RNA-seq has empowered many research areas and has led to new discoveries throughout the mRNA field [40]. In the present study, high-throughput transcriptome analyses were employed to study the differential gene expression profiles of three different follicles in different laying stages.

DEGs were significantly enriched in the extracellular matrix, extracellular region, extracellular region part, extracellular space, ECM receptor interaction, collagen containing extracellular matrix, and collagen trimer. The abilities of ECM to direct cell proliferation, differentiation, and function imply its remodeling in normal ovarian function [41]. The wall of the hen follicle is mainly composed of the extracellular matrix (ECM), which comprises collagenous fibers, dermatan sulfate, heparan sulfate, elastin, and hyaluronic acid [42].

Protein network interaction analyses of DEGs in W51 identified several genes associated with follicle development including *COL4A2*, *COL1A2*, *COL4A1*, *COL5A2*, *COL12A1*, *ELN*, *FBN2*, *ALB*, *MMP10*. COL4A2 (collagen type IV alpha 2 chain), COL1A2 (collagen type I alpha 2 chain), COL4A1 (collagen type IV alpha 1 chain), COL5A2 (collagen type V alpha 2 chain), COL12A1 (collagen type XII alpha 1chain) are five kinds of collagen. Type IV collagen is the main component of the basement membrane and constitutes its skeleton. It not only maintains the integrity of the basement membrane but also plays a key role in its formation. In normal conditions, the basement membrane is stable, dense, and continuous and can prevent macromolecules and cells from passing through [43]. The *ELN* gene encodes elastin. Fibrillin microfibrils are widely distributed components of extracellular matrices that function in the formation of elastin, serve structural roles and provide substrates for cell adhesion [44]. Albumin encoded by *ALB* may be a requirement for the control of follicle growth, which is attributable to albumin binding to specific cell-membrane components followed by the intracellular uptake of Alb-bound substances [45]. The *MMP10* (matrix metallopeptidase 10) gene belongs to the matrix metallopeptidase family. A growing body of evidence suggests that MMPs play a relevant role in the ECM remodeling of ovarian tissues [46–53]. Many MMPs are produced in the mammalian ovary and participate in the regulation of ovarian functions [46, 49, 51, 53–55]. It indicates that increased collagen may support the structural integrity of follicles during growth.

## Conclusions

The current study identified a series of key genes and signaling pathways associated with chicken follicular development by RNA-seq and bioinformatics analysis. These key genes (*COL4A2*, *COL1A2*, *COL4A1*, *COL5A2*, *COL12A1*, *ELN*, *FBN2*, *ALB*, *MMP10*) may regulate egg production by taking part in the extracellular matrix, extracellular region, extracellular region part, extracellular space, ECM-receptor interaction, collagen containing extracellular matrix and collagen trimer. The study constructed the transcriptional profiles of chicken growing follicles in different laying stages laying a foundation for further research on follicular development.

## Supplementary Information

**Additional file 1:** **Supplementary Table 1. **qPCR Primer sequences.

**Additional file 2:** **Supplementary Table 2. **Summary of sequencing reads mapping to the reference genome and quality parameters.

**Additional file 3:** **Supplementary Table 3.** Information of all enriched GO Terms based on DEGs of W51 intersection. **Supplementary Table 4.** Information of all enriched KEGG pathway based on DEGs in W51 intersection. **Supplementary Table 5.** Information of all enriched GO Terms based on DEGs in SWF intersection. **Supplementary Table 6.** Information of all enriched KEGG pathway based on DEGs in SWF intersection

## Data Availability

The RNA sequencing data used and analyzed during the current study are available from the NCBI (accession number: PRJNA795703 and the link of the website: http://www.ncbi.nlm.nih.gov/bioproject/795703).

## Abbreviations

RNA-Seq: RNA sequencing
SWF: Small white follicle
SYF: Small yellow follicle
LYF: Large yellow follicle
DEGs: Differentially expressed genes
GO: Gene Ontology
KEGG: Kyoto Encyclopedia of Genes and Genomes
QRT-PCR: Quantitative Real-time PCR
