# Supplementary material for: Grade follicles transcriptional profiling analysis in different laying stages in chicken
Source: BMC Genomics. 2022 Jul 7;23:492. doi: 10.1186/s12864-022-08728-w (PMC9260967; doi:10.1186/s12864-022-08728-w)
Supplement: Supplementary file 1 — Additional file 1: Supplementary Table 1. qPCR Primer sequences. [file 12864_2022_8728_MOESM1_ESM.doc]

**Supplementary table 1 qPCR Primer sequences**

| **Gene** | **Genbank accession no.** | **Primer sequence (5’-3’)** | **Amplification length (bp)** |
| --- | --- | --- | --- |
| SPP1 | NM_204535.5 | F: GGCATTTCTTTGCTTGTGCTTT  R: GGCTCCTGGGGTCGTATTT | 110 |
| FOXL2 | NM_001012612.1 | F: CATGAACAACTCGTGGCCG  R: GGCCCGAGAGTCCTTTCAC | 110 |
| CYP19A1 | NM_001001761.3 | F: TGCCAAAAGCACAAAAGGAAGTG  R: CTTCATTCTTGTGGATGGCATGAT | 102 |
| IGF1 | NM_001004384.3 | F: TGCCAAAAGCACAAAAGGAAGTG  R: CTTCATTCTTGTGGATGGCATGAT | 102 |
| *β-actin* | NM_205518.1 | F: TATTGCTGCGCTCGTTGTTG  R: GGGCGACCCACGATAGATG | 102 |

*Abbreviations: SPP1*, secreted phosphoprotein 1; *FOXL2*, forkhead box L2; *CYP19A1*, cytochrome P450 family 19 subfamily A member 1; *IGF1,*insulin like growth factor 1;*β-actin*, actin beta.
