# Supplementary material for: Grade follicles transcriptional profiling analysis in different laying stages in chicken
Source: BMC Genomics. 2022 Jul 7;23:492. doi: 10.1186/s12864-022-08728-w (PMC9260967; doi:10.1186/s12864-022-08728-w)
Supplement: Supplementary file 2 — Additional file 2: Supplementary Table 2. Summary of sequencing reads mapping to the reference genome and quality parameters. [file 12864_2022_8728_MOESM2_ESM.docx]

**Supplementary table 2. Summary of sequencing reads mapping to the reference genome and quality parameters**

| **Sample name** | **Raw reads** | **Clean reads** | **Q20(%)** | **Total mapped (%)** | | **GC content(%)** |
| --- | --- | --- | --- | --- | --- | --- |
| W22-SWF1 | 26,702,220 | 25,484,586 | 97.32 | | 92.57 | 52.65 |
| W22-SWF2 | 29,896,467 | 27,869,513 | 97.36 | | 91.17 | 53.51 |
| W22-SWF3 | 30,864,836 | 28,939,528 | 97.38 | | 91.21 | 53.05 |
| W22-SWF4 | 29,866,690 | 28,687,557 | 97.17 | | 92.57 | 52.42 |
| W22-SYF1 | 28,113,291 | 27,268,675 | 97.85 | | 92.92 | 52.85 |
| W22-SYF2 | 29,010,748 | 27,699,434 | 97.12 | | 91.18 | 53.3 |
| W22-SYF3 | 20,957,524 | 19,791,705 | 97.22 | | 92.31 | 52.55 |
| W22-SYF4 | 23,449,212 | 22,493,281 | 97.7 | | 93.36 | 51.93 |
| W22-LYF1 | 24,399,869 | 23,662,156 | 97.7 | | 92.61 | 52.98 |
| W22-LYF2 | 20,576,346 | 19,555,937 | 97.39 | | 92.07 | 52.04 |
| W22-LYF3 | 24,135,800 | 23,108,586 | 97.32 | | 91.26 | 52.93 |
| W22-LYF4 | 23,808,352 | 22,764,112 | 97.37 | | 92.94 | 52.64 |
| W31-SWF1 | 23,215,207 | 21,583,125 | 96.75 | | 92.01 | 52.31 |
| W31-SWF2 | 31,607,379 | 29,643,777 | 97.29 | | 92.39 | 52.47 |
| W31-SWF3 | 26,790,628 | 26,106,972 | 97.84 | | 92.72 | 52.8 |
| W31-SWF4 | 27,963,741 | 26,706,915 | 97.13 | | 91.70 | 52.61 |
| W31-SYF1 | 19,594,624 | 18,911,563 | 97.45 | | 93.51 | 50.39 |
| W31-SYF2 | 24,937,511 | 23,905,641 | 97.32 | | 92.36 | 51.92 |
| W31-SYF3 | 21,541,046 | 20,653,241 | 97.46 | | 92.95 | 51.91 |
| W31-SYF4 | 20,151,246 | 19,260,089 | 97.34 | | 92.34 | 51.8 |
| W31-LYF1 | 35,470,696 | 34,680,085 | 97.78 | | 91.95 | 52.22 |
| W31-LYF2 | 25,246,328 | 24,163,967 | 97.32 | | 93.01 | 52.05 |
| W31-LYF3 | 23,374,565 | 22,298,984 | 97.44 | | 92.93 | 52.93 |
| W31-LYF4 | 23,822,691 | 22,485,159 | 97.28 | | 92.01 | 53.56 |
| W51-SWF1 | 23,888,950 | 22,563,559 | 97.16 | | 91.97 | 53.11 |
| W51-SWF2 | 22,707,690 | 21,527,539 | 97.27 | | 92.27 | 53.05 |
| W51-SWF3 | 23,101,553 | 22,247,753 | 97.27 | | 92.43 | 52.02 |
| W51-SWF4 | 27,009,208 | 26,192,491 | 97.79 | | 92.70 | 52.77 |
| W51-SYF1 | 29,029,195 | 27,561,487 | 97.47 | | 92.84 | 51.4 |
| W51-SYF2 | 23,207,430 | 22,089,157 | 97.13 | | 92.28 | 53.03 |
| W51-SYF3 | 23,349,074 | 22,312,518 | 97.46 | | 92.75 | 52.12 |
| W51-SYF4 | 22,611,822 | 21,163,906 | 97.25 | | 92.65 | 52.46 |
| W51-LYF1 | 21,380,055 | 20,201,027 | 97.08 | | 92.55 | 52.51 |
| W51-LYF2 | 22,047,980 | 20,901,393 | 97.23 | | 92.26 | 53.41 |
| W51-LYF3 | 21,315,875 | 20,440,530 | 97.36 | | 92.45 | 52.71 |
| W51-LYF4 | 22,828,142 | 21,353,779 | 97.31 | | 92.50 | 53.14 |
