# Supplementary material for: Grade follicles transcriptional profiling analysis in different laying stages in chicken
Source: BMC Genomics. 2022 Jul 7;23:492. doi: 10.1186/s12864-022-08728-w (PMC9260967; doi:10.1186/s12864-022-08728-w)
Supplement: Supplementary file 3 — Additional file 3: Supplementary Table 3. Information of all enriched GO Terms based on DEGs of W51 intersection. Supplementary Table 4. Information of all enriched KEGG pathway based on DEGs in W51 intersection. Supplementary Table 5. Information of all enriched GO Terms based on DEGs in SWF intersection. Supplementary Table 6. Information of all enriched KEGG pathway based on DEGs in SWF intersection [file 12864_2022_8728_MOESM3_ESM.docx]

Supplementary table 3 Information of all enriched GO Terms based on DEGs of W51 intersection

| ONTOLOGY | GO Terms | DEGs No. | P-adjust | Genes |
| --- | --- | --- | --- | --- |
| CC | extracellular matrix | 4 | 0.000519 | COL1A2/COL12A1/ELN/CCN3 |
| CC | collagen-containing extracellular matrix | 3 | 0.001818 | COL1A2/COL12A1/ELN |
| CC | extracellular region part | 5 | 0.002353 | ALB/COL1A2/COL12A1/ELN/CCN3 |
| CC | extracellular region | 5 | 0.005991 | ALB/COL1A2/COL12A1/ELN/CCN3 |
| CC | collagen trimer | 2 | 0.006119 | COL1A2/COL12A1 |
| CC | supramolecular complex | 3 | 0.016773 | COL1A2/ELN/TUBB3 |
| CC | supramolecular polymer | 3 | 0.016773 | COL1A2/ELN/TUBB3 |
| CC | supramolecular fiber | 3 | 0.016773 | COL1A2/ELN/TUBB3 |

Supplementary table 4 Information of all enriched KEGG pathway based on DEGs in W51 intersection

| Pathways | DEGs No. | P-adjust | Genes |
| --- | --- | --- | --- |
| ECM-receptor interaction | 3 | 0.002121 | COL1A2/COL4A2/COL4A1 |
| Focal adhesion | 3 | 0.011734 | COL1A2/COL4A2/COL4A1 |

Supplementary table 5 Information of all enriched GO Terms based on DEGs in SWF intersection

| ONTOLOGY | GO Terms | DEGs No. | P-adjust | Genes |
| --- | --- | --- | --- | --- |
| BP | acute inflammatory response | 3 | 0.021 | EXFABP/TF/IL6 |
| BP | response to bacterium | 5 | 0.021 | EXFABP/LYZ/AVD/TF/IL8L2 |
| BP | antimicrobial humoral response | 3 | 0.021 | AVD/TF/IL8L2 |
| BP | immune response | 6 | 0.027 | EXFABP/AVD/TF/IL6/BLB3/IL8L2 |
| BP | defense response | 6 | 0.035 | EXFABP/LYZ/AVD/TF/IL6/IL8L2 |
| BP | humoral immune response | 3 | 0.035 | AVD/TF/IL8L2 |
| BP | response to lipopolysaccharide | 3 | 0.035 | EXFABP/TF/IL8L2 |
| BP | response to external biotic stimulus | 5 | 0.035 | EXFABP/LYZ/AVD/TF/IL8L2 |
| BP | response to other organism | 5 | 0.035 | EXFABP/LYZ/AVD/TF/IL8L2 |
| BP | response to biotic stimulus | 5 | 0.035 | EXFABP/LYZ/AVD/TF/IL8L2 |
| BP | response to molecule of bacterial origin | 3 | 0.037 | EXFABP/TF/IL8L2 |
| BP | multi-organism process | 6 | 0.041 | EXFABP/LYZ/AVD/TF/IL8L2/ASTL |
| BP | regulation of cartilage development | 3 | 0.041 | EXFABP/SCX/GREM1 |
| BP | inflammatory response | 4 | 0.046 | EXFABP/TF/IL6/IL8L2 |
| CC | extracellular space | 8 | 0.005 | COL9A1/EXFABP/LYZ/TF/GREM1/IL6/SCT/IL8L2 |
| CC | extracellular region part | 8 | 0.005 | COL9A1/EXFABP/LYZ/TF/GREM1/IL6/SCT/IL8L2 |
| CC | extracellular region | 9 | 0.005 | COL9A1/EXFABP/LYZ/AVD/TF/GREM1/IL6/SCT/IL8L2 |

Supplementary table 6 Information of all enriched KEGG pathway based on DEGs in SWF intersection

| Pathways | DEGs No. | P-adjust | Genes |
| --- | --- | --- | --- |
| Cytokine-cytokine receptor interaction | 9 | 0.000255 | CXCL13/CXCR1/CCL19/CCL4/LOC100857191/IL6/CXCR5/IL1R2/IL8L2 |
